# Using Social Media to Discover Public Values, Interests, and Perceptions about Cattle Grazing on Park Lands

**DOI:** 10.1007/s00267-013-0216-4

**Published:** 2013-12-17

**Authors:** Sheila J. Barry

**Affiliations:** Division of Agriculture and Natural Resources, University of California Cooperative Extension, 1553 Berger Drive, Building 1, San Jose, CA 95122 USA

**Keywords:** Cows, Public land grazing, Social media, Recreation, Photo-sharing, Endangered species

## Abstract

In the western United States, livestock grazing often co-exists with recreation, cultural resource management and biodiversity protection on federal and state protected rangelands as well as on many local government open space areas. While the value of livestock grazing for managing rangeland vegetation to reduce fire fuel loads and improve wildlife habitat is increasingly recognized by resource management professionals, public concerns, and conflict between recreationist and livestock have led to reductions in public land grazing. Traditional public input methods yield a constrained picture of people’s attitudes toward cows and public land grazing. Public meetings, hearings, and surveys, the most commonly used mechanisms for public land managers to solicit public opinion, tend to foster participation of organized special interests or, in the case of surveys, focus on a specific topic. General public input is limited. This study explored the use of personal photography in social media to gain insight into public perceptions of livestock grazing in public spaces. Key findings of this study include that many recreationist in grazed San Francisco Bay Area parks shared views, interests, and concerns about cows and grazing on the photo-sharing website, Flickr^TM^ that seldom show up at a public meeting or in surveys. Results suggest that social media analysis can help develop a more nuanced understanding of public viewpoints useful in making decisions and creating outreach and education programs for public grazing lands. This study demonstrates that using such media can be useful in gaining an understanding of public concerns about natural resource management.

## Introduction

In the western United States, livestock often grazing co-exists with recreation, cultural resource management, and biodiversity protection on most federal and state administrated rangelands as well as on many local government open space areas (Resnik et al. 2006). On these public rangelands, grazing is part of a working landscape which provides fire fuel reduction, wildlife habitat, and biodiversity, and protects historic land uses and rural character (Huntsinger et al. 2007). Although many concerns about grazing have been addressed in the western United States with grazing plans, improved grazing management, monitoring, and better understanding of grazing’s ecological role (Briske 2011), there are still indirect impacts that may be considered negative, such as trampling, livestock waste, and grazing infrastructure, introduction of invasive species, and greenhouse gas emissions (Huntsinger et al. 2007; Ringgold 2009). In addition, potential conflicts between livestock and park users are worrisome for land managers, livestock operators, and park users on public lands (Huntsinger et al. 2007; Resnik et al. 2006; Ringgold 2009). These concerns and conflicts have led some public land managers to limit or curtail the use of grazing on the lands they manage.

While decisions to limit or curtail grazing on public lands are based partly on minimizing negative visitor experiences such as periodic scares and rare direct injuries (Tempest 2004), they also may be based on a belief that public opinion is predominantly negative toward grazing (Nardi 2009). For example, although cattle had grazed two city parks for weed abatement in the City of Walnut Creek for decades (Nardi 2009), in 2009 city officials decided to end grazing in these parks. The officials used results from a visioning process which included public workshops and surveys to conclude that the public was overwhelmingly negative toward grazing on city park lands, basing their decision partly on park users’ complaints of cattle trampling trails and “increases in attacks by cattle on dogs and people.” However, just 1 year after grazing was removed from the two parks, neighboring homeowners petitioned the city to return the cows because of their concerns for catastrophic wildfire (Rieber 2011). In response to the risk of catastrophic wildfire, city officials provided some weed abatement with fire breaks created by fee-for-service goat grazing. Residents still miss the cattle grazing which provided more extensive vegetation management and revenue to the city (Nardi 2012).

In terms of recreationists’ opinions, previous studies have shown that their expectations for public lands affect their acceptance of grazing. For example, Sanderson et al. (1986) found that the more experience recreationists had on grazed lands, the less likely they were to have negative perceptions of grazing. Brunson and Gilbert (2003) documented that in Utah’s Grand Staircase Escalante National Monument, hikers were more likely to feel negatively toward livestock use than hunters. Research has also shown that there is a rural–urban divide in general environmental attitudes and beliefs toward grazing (Howell and Laska 1992), especially when the rural economy depends on rangelands (Brunson and Steel 1996).

Little is known about the attitudes, beliefs, and interests of a largely urban public recreating on neighboring grazed park and open space lands. Public land managers and decision makers seeking to understand public viewpoints in order to aid in decisions usually hold public meetings or conduct surveys. The California Environmental Quality Act (CEQA) (Remy et al. 1999) and the National Environmental Policy Act (NEPA) (Moorman and Ge 2006) both require public hearings and solicitation of public comments for public planning and management decisions. While these processes ensure that public agencies receive and evaluate public reaction to the environmental consequences of their actions, they tend to favor negative feedback and may not accurately identify or address broad public beliefs or interests (Moote et al. 1997). These processes also do little to facilitate further public discourse or educate the public to develop well-informed opinions. There is growing recognition that more deliberative process which advances public debate, public reflection, and the development of informed public opinion is essential to address the complex issues related to management of natural resources including public open spaces (Schusler et al. 2003, Parkins and Mitchell 2005). Understanding the values, interests, and perceptions of recreationist and local communities toward grazing and cattle to develop an effective outreach effort would be the first step to a deliberative process that results in the successful management of grazed park and open space lands (Resnik et al. 2006).

This study considers the use of data generated from Flickr™, a photo-sharing social media website, as an alternative way to gain insight into public values, attitudes, and concerns about cattle and grazing on park and open space lands. Offered apart from the public meeting or hearing setting, where the focus is usually on contentious decisions, and outside of a survey, where questions both lead and constrain response, photos, and opinions on Flickr™ are public perceptions volunteered as the photographers reflect on their experience and respond to their online communities. We might expect viewpoints to emerge that are less a result of current polemics and more an unfettered response to experience. Comments and photos posted on Flickr™ are used to address the following questions:

- When people visit public lands with grazing livestock present, what do they photograph?
- How do park users respond when they encounter and choose to photograph something seemingly undesirable or potentially frightening, such as manure, a rutted trail, or cows on the trail?
- When people take photos or look at other people’s photos from public lands that have been tagged with cow(s) or grazing, what kind of comments do they make?
- How do comments from photos taken on public lands, tagged with cow(s) or grazing, compare to comments from photos taken on a nearby ungrazed public land?
- How do comments from photos taken on public lands, tagged with cow(s) or grazing, compare to comments from photos of other subjects that may be considered environmentally negative or frightening, such as smog or snakes?

### Conceptual Context

With the exponential growth in social media and the willingness of people to share their ideas via internet communities, there is a growing interest in what we can glean from social media (O’Connor et al. 2010). Research to date has focused on extracting public opinion from text-based social media sites such as Twitter and Facebook (Mehta et al. 2012; Agarwal et al. 2011; O’Connor 2008) and has yielded mixed results. Twitter content analysis was found to replicate consumer confidence and presidential job approval polls (O’Connor et al. 2010). However, another study reported by the Pew Research Center (2013) found that opinions expressed on Twitter on politics and social policies differed from public opinion based on topic, sometimes more liberal, sometimes more conservative, and often more negative. Researchers concluded that Twitter users are not representative of the general public but reflect “the narrow sliver of the public” using Twitter and an even narrower slice in those tweeting on a particular subject. Although the value of social media text for replacing traditional methods of evaluating public opinion may be limited, its value in adding context to public conversation has been demonstrated (Leyden 2012). In addition, the exponential growth in use, types, and connections between various social media platforms as well as the amount of research focused at extracting query-driven data will continue to improve its value in understanding public viewpoints (O’Connor et al. 2010).

Visual social media, the sharing of photos and video, has received limited attention regarding its ability to extract information about social values and interests. Even before social media, photographs have been recognized as a resource for visual narratives about society and culture (Harrison 2004). Harrison (2004) explored the social dimensions of “everyday” or amateur photography. She concluded that what is worthy of being photographed, displayed, or stored reveals choices that confirm values, social relationships, and identifies. She also noted that in western societies “everyday” photography centers around family, tourism, and leisure or recreation. Social media photo-sharing websites, like Flickr™^,^ allow photographers to share, tag, and comment on photos, creating a source of data about values, interests and perceptions, especially for topics covered in “everyday” photography. Flickr™ is an image storing and sharing service that offers limited use for free and unlimited use for a modest fee. This service allows people to title, tag, and describe their images, and allows viewers to comment on images by others. During the time frame of this study (2002–2009), Flickr™ had up to 27.5 million visitors to the site per month. The users were evenly divided by gender and more than 70 % had some college education. The age breakdown of the users make it one of the more matured social networking sites, as less than 20 % of the users were under the age of 24, 40 % were between 25 and 44, and 40 % are over the age of 45 years. During the study period there were more than 3 billion photos hosted on the Flickr™ site (New Media Lab 2008).

Past efforts to learn from Flickr™ data have focused largely on tagging (Marlow et al. 2006) and geospatial information (Kennedy et al. 2007). Recent efforts have built on earlier work on understanding the social use of personal photography (Harrison 2004) and, now, image-sharing (Van House 2007). Flickr™ data are derived from personal photographs of everyday activities that are taken by “ordinary” people and shared in a public forum, which means Flickr™ provides an opportunity to learn about the values, interests, concerns, and perceptions of park users, their friends, and others who are interested in parks.

### Study Area

Over 13 million hectares or 30 % of California is public land that is classified as rangeland (CDF-FRAP 2010). The opportunities for public outreach about grazing are probably no greater than in the geographic area for this study, the San Francisco Bay Area, where public grazed rangelands are managed by more than 20 public entities from the local to national level (Barry 2004). Recreation, including hiking, biking, dog walking, horseback riding, and hang gliding, occurs across 54,000 ha of the grazed public land (Barry and Amme 2009). Grazed parks in the East Bay Regional Park District (EBRPD), the largest steward of publically held land in the San Francisco Bay Area, are visited by over 2.5 million visitors per year (East Bay Park District unpublished attendance data for 2006 and 2007).

On these public rangelands, livestock grazing is accepted and often defended as an essential tool to manage vegetation (Sulak and Huntsinger 2007). While management objectives vary, they typically use grazing for fuel reduction or vegetation management to improve habitat for native plants and animals, including several endangered species (Huntsinger et al. 2007). Despite considerable evidence of the benefits of grazing for numerous endangered species in California (Hayes 1998; Warrick and Cypher 1998; Weiss 1999; Marty 2005; Fellers and Kleeman 2007; Germano et al. 2012) it remains controversial. Lawsuits by environmental groups and park users still challenge some grazing leases and result in reduced or curtailed grazing (i.e., Los Padres Forest Watch et al. vs. California Department of Fish & Game 2010). Thus, social acceptance of grazing on California’s public rangelands presents both a challenge, requiring that common misconceptions be overcome and that grazing be well managed, and an opportunity to educate people about grazing, livestock, and food production.

Despite the frequent use of grazing and the controversies surrounding it, recent telephone and on-line surveys, where 400 and 6,294 participants, respectively, provided their opinions to the EBPRD for a master plan update, provided little information about public views of grazing (Strategic Research Institute 2011 unpublished). Cattle grazing was not addressed in the multiple choice questions, and of 1,631 comments from open ended questions, only 10 comments mentioned grazing. The comments included trail damage by cattle, requests that grazing be better monitored or managed, and/or review of grazing policies. Two requested that grazing cattle be removed from parks (East Bay Regional Park District 2013 and unpublished comments). This study seeks to gain a more nuanced understanding of people’s relationships with cattle grazing on public land through social media, one that could used by public land managers to create outreach and education programs and guide decision making.

## Methods

Data sets were developed from photos and associated comments posted on Flickr™ from February 2002 to October 2009. They were derived from searching photo titles, tags, and comments for location terms, such as park names, and subject terms, such as cow(s) and grazing (Table 1).

**Table 1:** Data sets developed from Flickr™

Data sets	Search terms		Number of photos	Number of photographers	Number of comments
	Location(s)	Subject(s)			
Grazed regional parks	33 park or place names in Alameda, Contra Costa, and Santa Clara counties^a^	Cow, cows, grazing	1,087	328	956
Grazed national park	Pt Reyes National seashore	Cow, cows, grazing	50	27	52
Ungrazed state park	Mt Diablo State Park	State park	50	35	58
Smog	California	Smog	50	47	71
Snakes	California	Snake, snakes	50	41	81
Rattlesnakes	California	Rattlesnakes	50	44	122

^a^ Anthony Chabot, Bishop Ranch, Briones, Black Diamond Mine, Dublin, Brushy Peak, Carquinez, Shoreline, Contra Loma, Coyote Lake, Cull Canyon, Del Valle, Diablo Foothills, Don Castro, Dry Creek, East Bay, Ed Levin, Garin, Grant Ranch, Harvey Bear, Lake Chabot, Las Trampas, Livermore, Ohlone, Pleasanton Ridge, Mission Peak, Morgan Territory, Rancho Canada del Oro, Round Valley, Sibley Volcanic, Sunol, Sycamore Valley, Tassajara, Wildcat

Photo information recorded for each data set included photo date, posting date, photographer’s name, photo name, photo comments by photographer, posted comments by others, and commenters’ names. Photo titles and all comments for each data set were also categorized as follows: photo quality comment, descriptive comment, positive comment, negative comment, and fearful comment. Fearful comments included those not based on an actual event, and fear experienced as a result of a described event. Coding images by categories is a method of content analysis which was originally developed to interpret written and spoken text, but has been adapted to be used on visual content (Gillian 2007). Categories for photo data sets presented in this study were determined only by assessing associated written content—photo titles and comments. Comments on photo quality were not included in the results or analysis nor are they discussed in this study. The “grazed regional parks” data set included every photo found under the location and subject search terms listed in Table 1.

**Fig. 1:**
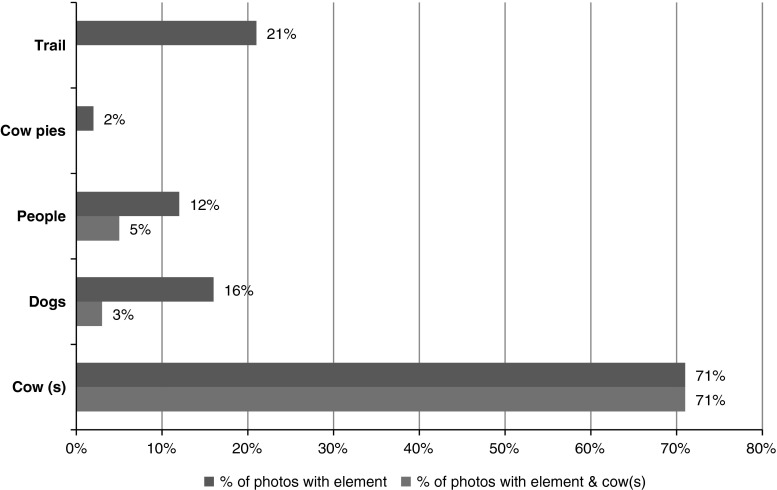
Percent from 1,087 photos in the “Grazed Regional Parks” data set with these elements in the photo

Every photo in the “grazed regional parks” data set was also reviewed with a content analysis approach. The presence of a cow(s), dog(s), people, trail(s), and manure was recorded for each photo and the frequency of each recorded. The frequency of certain visual images e.g., cow(s), manure, trails, and dogs was recorded for each image after a visual assessment following methodology described by Gillian (2007) (Fig. 1). Additional data sets, “grazed national park,” “ungrazed state park,” “snakes,” “rattlesnakes,” and “smog” were created for comparison. The grazing-related data sets were used to compare the frequency of different categories of comments on grazed and ungrazed parks. Comments on snakes and rattlesnakes were used to compare the frequency and content of comments expressing fear with those expressing fear of cattle, under the assumption that being fearful of snakes and especially rattlesnakes is common, and encounters are known to happen on local park lands. Smog was selected as a data set to compare the frequency of negative comments under the assumption that smog is a common occurrence in the parks that is widely considered negative. To create these data sets, 50 photos with associated comments were randomly selected from data sets created by specific search terms (Table 1). For comparing each category of comments among the 6 data sets, Fisher’s exact test (FET) was performed followed by Holm’s adjustment for pairwise comparison using the “fmsb”package (Nakazawa 2013) of the statistical software R (R Core Team 2012).

## Results

First the outcomes of the content analysis of the “grazed regional parks” data set are reported. All photos from these parks tagged with “grazing” or “cow(s)” totaled about 1,087 photos (Table 1). Of these, 733 photos included comments, and there were 956 comments categorized.

### What did people who posted photos from the grazed regional parks take pictures of?

**Fig. 2:**
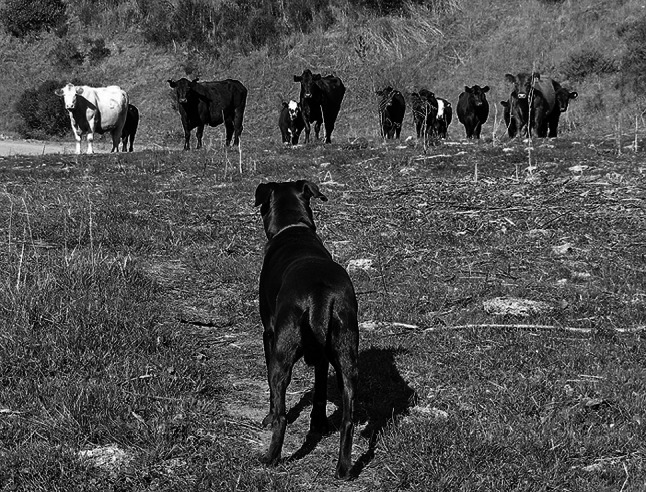
“Moment of Truth—and she was face to faces with this small herd…” Photo and comment by Flickr™ user, Doug Greenberg

Since the search terms included the tags of cows, cow and/or grazing, a strong majority, 71 % of the 733 photos used in the comment analysis included a cow or cows (Fig. 1). Some photos captured pictures of the photographer, friends or a dog with a cow or cows. Many of the photos with people and a cow or cows featured the subjects posing in front of the animal(s). Photos with cows and dogs included scenes with dogs chasing cattle, dogs standing their ground against a cow or group of cows (Fig. 2), and groups of dogs being walked.

### What kind of comments are associated with the photos of grazed regional parks?

Of 956 comments from photographers or online viewers (Table 1), 71 % were categorized as descriptive (Table 2). These comments described the location, event, date, or landscape, but with no obvious opinion about cows or grazing, including:

**Table 2:** Comments by category for the 6 data sets used in the study, and the proportion of each type of comment in each data set

Data set	Positive comment	Negative comment	Fearful comment	Descriptive comment	Total
Grazed regional parks	222 (23.2 %)^a^	14 (1.5 %)^a^	46 (4.8 %)^a^	674 (70.5 %)	956
Grazed national park	12 (23.1 %)^a^	0^a^	2 (3.8 %)^a,b^	38 (73.1 %)	52
Ungrazed state park	9 (15.5 %)^a,b^	0^a^	0^a^	49 (84.5 %)	58
Smog	3 (4.2 %)^b^	16 (22.5 %)^b^	0^a^	52 (73.2 %)	71
Snakes	15 (18.5 %)^a,b^	0^a^	11 (13.6 %)^b^	55 (67.9 %)	81
Rattlesnakes	19 (15.6 %)^a,b^	1 (0.8 %)^a^	54 (44.3 %)^c^	48 (39.3 %)	122
Total	280	31	113	916	1,340

Total is for all comments for photos in each data set

Different letters within a column indicate a significant difference between proportions of comments in a comment category (*P* < 0.05 Fisher’s exact test with Holms adjustment)

Lots of wildflowers and cows. Hello tiny cows on the hillside.

Taken at Lake Del Valle.

Cow pool party. (Shows a livestock pond encircled by cows).

A cow grazing….

Cow munching on some grass near the lake.

Some descriptive comments illustrated the commenter’s lack of knowledge regarding cows and grazing:

I don’t know why, but I thought cows in California were kept indoors.

I never knew what these cows were doing here.

While other descriptive comments described potential interactions with dogs and cattle:

I bet he would like to teach them how to run or at least test their stamina.

I am a herding dog. Let me go.

A little over 23 % of comments were positive toward cows and grazing including:

Wonderful to see cows being just cows and happy ones.

Superb…love the cow.

Oh, I just love fuzzy winter cows.

Happy cows eating grass not corn.

I love this little guy.

Cows happily range over the lands of Sunol Regional Wilderness keeping down the fuel load.

The sign said that cows have been known to nudge hikers when startled. It’s hard to picture a cow nudging a hiker. I doubt that would be the adjective I would use to describe it if I saw it happen. Generally they are scared of hikers and actually help to spread seeds, control non-native plants, and overall keep a healthy preserve. I kind of like sharing the green hills with them.

I couldn’t help but notice the beautiful scenery. (Cows were included in this photo).

Beautiful spring at Morgan Territory. Green grass, clouds, and the cows.

As much as I struggled over the steep hills on this hike, all the grazing cattle and howling coyotes made it worth the sweat.

I went on a really long hike and saw some cool things from meadows to steephills to trees, cows, and interesting rock forms.

Less than 2 % of the comments were negative, and these focused on the presence of cows and/or manure rather than on grazing (Table 2). Negative comments included:

It’s a little anti-climatic when you hike uphill for 2 h and see a herd of cows upon arrival.

However, this kind of landscape also attracts a lot of cows which seem to have more privileges than me in roaming around.

The only downside was/is that there are cows grazing there a lot and hence: cow patties! Many of the dogs had a taste and all rolled in cow poop quite thoroughly. (This comment was posted by a self-identified professional dog walker.)

**Fig. 3:**
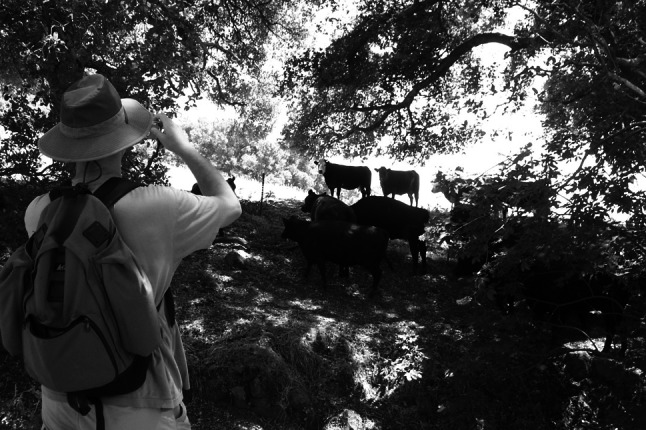
“Making peace with cows—they seem to be leaving us.” Photo and comment by Flickr™ user, Daniel Cooke

Fear of the cattle was expressed in a bit less than 5 % of the comments (Table 2; Fig. 3), including:

I try to conquer my fear of cows by photographing them.

Watch out for those cows.

Got close to this cow for this shot. You can see she’s giving me the stink eye here so I put the camera away.

The cows scared us to death.

Beware! Mad cows!

This is the cow that blocked our path! Would you want to cross him?

I told them that I’m a vegetarian and they let me go.

He wasn’t too keen about being photographed. In addition to the unfriendly stare, he made menacing noises.

A cow that was not happy to see us and almost chased us.

We turned back here as the cow was on the trail path.

We turned around when we were faced with the option of having to walk right through a herd of cows.

Seven comments, less than 1 % of all comments, included a description of an actual event with “aggressive” animal, such as being chased, including:

Ahh, we were chased last weekend by a young male-err.

At least these cows didn’t chase us like last week’s did.

Happy cows may come from California, but bored cows come from Fremont. I actually tried to have a picnic, but then a bull comes charging us. We got up and ran for our lives. Our bread, cheese, and blanket didn’t make it.

### Comparative Analysis

The next set of results are based on comparisons among all the data sets: “grazed regional parks,” “grazed national park” (Point Reyes National Seashore), “ungrazed state park” (Mount Diablo State Park), and “Snakes,” “Rattlesnakes,” and “Smog.”

### How do comments from photos taken on grazed regional parks compare to comments from photos taken on a nearby grazed or ungrazed public land?

There was no significant difference in the proportion of positive, as opposed to negative and descriptive, comments for grazed and ungrazed parks (*P* < 0.05 FET). There were positive comments on 23 % of photos tagged for cow(s) or grazing in the regional parks, 23 % of 50 randomly selected photos with tags for cows or grazing and comments from the grazed national park, and about 16 % of 50 randomly selected photos from the ungrazed state park (Table 2). The proportion of negative comments posted for grazed regional parks is very small (<2 % negative), while no negative comments were posted for the other grazed and ungrazed parks. Though not statistically significant, the one difference in comment types between grazed and ungrazed parks is that there were, of course, no comments reflecting fear of cows in the ungrazed park, while there were about 5 % in the grazed regional park and about 4 % in the grazed national park (Table 2).

Similar to the positive comments for the “grazed regional parks,” positive comments from the “grazed national park” tended to focus on the cows and the pastoral landscape, such as:

Moo…like I said, Pt Reyes has dairy farms. In some areas, you hike right through the fields with the cows; in other places, people and cows are separated. In fact, many parks in this part of California “double” as grazing land—it’s a great way to control grass growth and eliminate fire hazards. So cattle on trails are common.

This photo just does not do this place justice. This is a view of part of the National Seashore in Pt Reyes, toward Drake’s Beach. Most of the land is organic grass-fed beef farms (these cows are in heaven), the water in the center is used to farm oysters (also heavenly).

Happy cows take a leak.

Positive comments from the “ungrazed state park” also largely focused on the landscape, including:

Mt Diablo State Park is a wonderful place. In the spring there are wildflowers, lots of green vegetation, and brown colored grasses.

A pretty, sunlit meadow at Mt. Diablo State Park framed by an older oak tree.

### How do comments from photos taken on public lands and tagged with cow or grazing compare to comments from photo comments in California about other subjects common in the parks that people may consider environmentally negative or frightening, such as smog or snakes?

Photographers and other commenters who commented about smog on Flicker™ made a significantly higher proportion of negative comments toward smog, about 23 % of all comments on photos with a smog tag, than those who commented on cows or grazing in the Grazed regional parks, less than 2 % of photos with a cows or grazing tag (*P* < 0.05 FET) (Table 2). The 4 % positive comments associated with smog mentioned the “beautiful sunsets” created by the smog (Table 2), and is a significantly lower proportion of positive comments than that for cows or grazing in the regional parks (23 %) or national park (23 %), but not a significantly lower proportion of positive comments than in the ungrazed state park (16 %) (*P* < 0.05 FET).

About 44 % of comments for photos tagged with the term “rattlesnake” were fearful, significantly more than the than 14 % of fearful comments on photos tagged with just snakes, 5 % of regional park photos tagged with cows or grazing, or 4 % of national park photos tagged with cows or grazing (*P* < 0.05 FET) (Table 2). Photos in the “Snake” data set were predominantly of non-venomous California king snakes and gopher snakes, yet fear was indicated by a significantly greater proportion of commenters from photos tagged with snakes, 14 %, than to the 5 % of fearful comments on photos tagged with cows or grazing in the grazed regional parks (*P* < 0.05 FET). The proportion of positive comments for these wildlife species was not significantly different in frequency to those for cattle or grazing.

## Discussion and Implications

The specific aims of this study were to explore the use of social media (Flickr™) to get a better understanding of the values, interests, and perceptions of recreationists about cattle grazing in parks and open space lands. A clear and more nuanced understanding of public viewpoints toward cows and livestock grazing is critical to successful public outreach and to the management of grazed park and open space lands. Because hearings, surveys, and public comments generally provide insight limited to specific issues and tend to favor negative feedback (Moote et al. 1997), these methods may not accurately identify public values and interests and provide limited insight to develop public outreach and education.

Indeed, while many public land managers assume based on traditional public input methods that recreationists uniformly oppose grazing in parks, the results of this study suggest otherwise. Key findings of this study include that many recreationists in grazed San Francisco Bay Area parks shared positive views about cows and grazing on Flickr™. While only a minority of users shared negative views about cows and grazing, these concerns were generally specific to the presence of manure and the fear of cows.

### Understanding Flickr™ Users

To understand why Flickr™ can yield different results from traditional public input methods, it is helpful to compare the people in the two groups. Public participation processes tend to foster participation of organized special interest groups while limiting participation by the general public (Facaros 1989; Fortmann and Lewis 1987). In addition, public processes like those required under NEPA and CEQA are generally geared to address specific management questions or decisions (Remy et al. 1999; Moorman and Ge 2006). In contrast, Flickr™ draws people who are from the general public and who have very different motivations. Notably, people use Flickr™ to express themselves and to share their experiences with others, rather than to argue one side or another of an environmental decision.

Van House (2007) identified four uses of personal photography that describe why people post pictures and comments on Flickr™ and are useful for understanding the findings of this study.

1. *Memory, narrative, and identity* Personal photos help to create memories about where people have been and what they did. These memories are critical to constructing a personal story and sense of identity. In this study, people’s efforts in recording a memory and creating a narrative are illustrated by the descriptive comments that represented the majority of those associated with photos in park data sets.
2. *Relationships* Photos with people and shared places and activities also develop a personal story. They reflect and reinforce relationships associated with the story. In this study, these relationships are likewise reflected in descriptive comments associated with the park data sets.
3. *Self-representation* Some people use photos and their comments to present themselves in a way they wish to be seen by others including the public. Some people are interested in posting on Flickr™ especially because it is a shared public forum. Although comments associated with self-representation could be simply descriptive in nature, they also provide an opportunity for people to express their values, opinions, and concerns. In this study, self-representation is evident in people’s indications of fears about cattle, snakes, and rattlesnakes. In some instances, they share their desire to overcome their fear of cows through, for example, taking pictures of cows or being photographed with cattle in the background.
4. *Self-expression* Both comments and photos provide an opportunity for people to reflect their unique point of view, creativity, or aesthetic sense. The use of Flickr™ and other social media for self-expression may tend to skew the photos and comments in a positive direction. However, it was evident from this study that when people photograph or comment on something that is widely considered negative (such as smog) or scary (such as rattlesnakes), these shared opinions are expressed to a significant degree in comments. On the other hand, commenters also found reasons to be positive about both snakes (some people like them) and smog (it can enhance a sunset).

While Flickr™ does store images, most users see the service as a social site for sharing a stream of their experiences. This includes ordinary snapshots of their day-to-day lives as well as exceptional images. Because users rarely go back to look at the images in their streams, tagging, titles, and comments are done almost exclusively for other viewers. Users are most likely to tag images that they think will be of particular interest to other viewers (Van House 2007). Perhaps most important for understanding the findings of this study, by drawing from people who simply want to share rather than those with a political goal, Flickr™ comments offers an opportunity to capture a diversity of public values, interests, and concerns toward cattle grazing in parks and open space lands among those who recreate there.

### Most Cow-Related Comments Were Positive

Park users whose photo tags, titles, or comments indicated awareness of being in a grazed park were generally positive about cattle and grazing. Of the 31 % of comments that expressed an opinion about cows, over 77 % were positive. Overall, only 2 % of comments were negative about cows and/or manure, and, it is worth noting that these comments were not negative about grazing itself. Moreover, for photos tagged with “cow” or “grazing,” very few photos included negative aspects of cattle, such as manure or rutted trails.

While the finding of positive views about cows and grazing is notable, even more useful are insights into the reasons behind specific opinions. The positive comments expressed user’s enjoyment of the pastoral scene and their recognition of cows grazing as “happy cows.” The message that cattle grazing reduces fire risk and enhances wildflowers was also clearly expressed by some commenters. The negative comments make it clear that some park users, especially those with dogs, are bothered by manure.

### Fear of Cows

Although very few comments or photos expressed negative comments about cows or grazing, some park users did share their anxiety about sharing the site with cattle: about 5 % of comments in the Regional Parks expressed fear of cows. However, these users also often stated a desire to overcome this fear, for example, commenting that, “I photograph cows to conquer my fear.” Others seemed uncertain about what could happen or how to respond. These comments not only illustrate the need for park user information on cattle behavior but also provide insight into the type of questions that should be addressed including what to expect, what are signs of aggression and how to respond.

### Management and Decision Making Opportunities

Managers may be able to overcome negative perceptions and fear of cows on public lands via education. One opportunity for educating the public is to explain why grazing is used as a management tool in parks. Some descriptive comments illustrate that some users do not understand why cattle are grazing park lands. Park users may be more tolerant of manure and rutted trails if more of them understand that grazing benefits park and open space lands by enhancing conservation of native habitats and species as well as by reducing fuels and thus the risk of fire.

Another opportunity for educating the public is to explain both cow production practices and the role that grazing on public lands plays in cattle production. Comments recognizing the connection between the grazing cattle and food production were largely absent. Understanding that local grazing benefits the local foodshed and related businesses may further increase recreationist acceptance of cows on public lands.

Finally, a more complete picture of public viewpoints, with insights gleaned from social media could help managers to identify and address conflicts between recreationists and cows in parks and open space lands. Although reports of injury are rare on San Francisco Bay Area grazed park lands (Barry and Amme 2009), concerns about liability and complaints about aggressive cattle need to be addressed. Some incidents are clearly related to livestock interactions with dogs and/or the cattle production cycle, as cattle may be particularly sensitive during calving time. The need to educate park users about both of these issues is evident in some of the photos. Some photos show dogs chasing cows while others show newborn calves, suggesting that the photographer may be too close to the calf and/or its mother.

Crafting an effective message about how to safely and comfortably recreate in a grazed park has proven to be challenging. The experience of the park user, and the temperament and differing activities of the cattle daily and seasonally, limit the ability of the parks to provide a “one size fits all” message to minimize conflict. For example, cattle behavior varies when they are resting, grazing, nursing, and traveling. Information gleamed from social media regarding people’s experiences and fears can help managers to identify the most essential and effective messages with which to tailor educational efforts for recreationists at particular grazed parks.

Park managers have a history of coping with wildlife, dog, and human interactions to draw on. Although it is likely that some recreationists will always find cows a bit scary, they are not the only animals that generate fearful comments. A significantly greater proportion of photos tagged with the terms “snake” or “rattlesnake” had fearful comments about them than did photos tagged with cows or grazing (Table 2). Explanations of the ecological value of a species and the need to behave carefully around some of them at different times of the year (including very common species such as deer!) have long been in the Park management portfolio.

### Research Needs

Openly increasing public participation in decision making processes regarding public lands can result in polarization (Moote, et al. 1997). The mainstream media (both print and commercial TV networks) are in the forefront of using social media to gauge public opinion, interests, and values, and increasingly derive data from public social media forums such as Twitter™ and Facebook™ (Pew Research Center 2013). Future efforts should look to integrate analysis from both text and visual social media, as multiple sources are likely to amplify specific viewpoints, interests, or values. Kennedy et al. (2007) concluded that community-contributed media and annotation can enhance our understanding of the world, but translating this understanding into data to inform public decisions remains a research need. With research findings that show how to collect, analyze, and translate data from social media, these new forms of expression could become an important tool to improve public policy.

## Conclusion

The overarching goal of this research was to explore opportunities in using social media as a means of obtaining an alternative view of perceptions of cattle grazing on public lands that is distinct from those that emerge in a polemical hearing or meeting, and broader than those provided by specific questions in a survey. The more nuanced viewpoints revealed in photo comments provide a basis for developing outreach materials and park policy. This study shows that Flickr™ can provide insight both through photos and comments into public perspectives on grazing in parks and open space lands. This work, however, is just a first step toward broadening this understanding, and additional research is necessary. Further analysis of social media may provide managers with broader insights into public opinion compared to those afforded by traditional methods on a wide range of issues important to park and open space management.
